# Congenital Anomalies in Neonates Admitted to a Tertiary Hospital in Southwest Ethiopia: A Cross Sectional Study

**DOI:** 10.4314/ejhs.v31i6.10

**Published:** 2021-11

**Authors:** Kassahun Birhanu, Workneh Tesfaye, Melkamu Berhane

**Affiliations:** 1 Department of pediatrics and child health, Jimma University, Jimma, Ethiopia

**Keywords:** Neonates, Congenital Anomalies, Folate, Jimma, Ethiopia

## Abstract

**Background:**

Congenital anomalies affect 2–3% of all live births. Anomalies of the central nervous system account for the highest incidence followed by that of the cardiovascular and renal systems. There is scarcity of data in developing countries like Ethiopia. The aim of the study was determining the magnitude and type of congenital anomalies and associated factors in neonates admitted to the neonatology ward of Jimma Medical Center, Southwest Ethiopia.

**Methods:**

Institution based cross sectional study was done from March 1 to July 30, 2020. A total of 422 mother-infant pairs were enrolled into the study. Structured questionnaires were used to capture the socio-demographic, obstetric and medical characteristics of the mothers, demographic characteristics of the infants and type of congenital anomalies. Univariate and multivariate logistic regression analyses were done and results are presented as narratives and using figures and tables.

**Results:**

Closer to one in five neonates admitted to the neonatology ward (78, 18.5%, 95%CI 14.7–22.3) had congenital anomalies; the majority (59, 13.98%) having only one type of anomaly. Anomalies of the nervous system (29, 6.87%) and gastrointestinal system (24, 5.68%) accounted for the majority of the cases. Inadequate antenatal care follow-up (p=0.018, AOR=1.9, 95%CI1.115, 3.257) and lack of folate supplementation during pregnancy (p=0.027, AOR=2.35, 95%CI 1.101, 5.015) were associated with congenital anomalies in the neonates.

**Conclusion:**

Congenital anomalies account for significant number of admissions. Significant association was seen between neonatal congenital anomalies and poor antenatal attendance and lack of folic acid supplementation during pregnancy.

## Introduction

The human reproduction is complicated process and is likely to be adversely affected by various factors related to the host and environment. The first trimester, especially between the third and eighth weeks of gestation, is crucial period of morphogenesis of organs. Any insult during this period can result in congenital Anomalies(1–3). Congenital anomalies affect 2–3% of all live births, the magnitude varying with geography, race and ethnicity(1–3). Even if all organ systems can be affected, anomalies of the central nervous system accounts for the highest incidence followed by that of the cardiovascular system and the kidneys(1–3).

The World Health Organization (WHO) estimates that around 270, 000 deaths worldwide (about 7% of all neonatal deaths) were caused by congenital anomalies in 2010(1). Few studies done in low and middle income countries (LMICs) have also indicated that congenital anomalies are prevalent problems in neonates(4–7). In the large proportion of the cases, the causes/risk factors of congenital anomalies are unknown even if genetic defects (like chromosomal anomalies), environmental factors (like exposure to teratogens), maternal chronic medical disorders (like diabetes mellitus), maternal infections (like rubella), and micronutrient deficiency (like folate deficiency) contribute significantly to the increased risk of congenital anomalies(2–4,6,8,9).

Few studies done in Ethiopia have demonstrated that anomalies of the nervous system account for the large proportion(10–14) followed by orofascial(12), musculoskeletal(11,12) and gastrointestinal(10) anomalies. Factors associated with congenital anomalies in Ethiopia were exposure to pesticides(11,13), consumption of khat(11,13), alcohol intake(13), maternal illnesses(13) and lack of folic acid supplementation during current pregnancy(13). The aims of this study are determining the magnitude and types of congenital anomalies and associated factors among neonates admitted to the neonatology ward.

## Materials and Methods

**Study area and period**: The study was conducted at the neonatology ward of Jimma Medical Center (JMC), a tertiary hospital in Southwest Ethiopia from March 1 to July 30, 2020. At the center, neonates younger than 15days are admitted to the neonatology ward whereas older neonates are admitted to the general pediatric ward.

**Study design**: Institution based cross-sectional study design was used to recruit participants consecutively until the required sample size was obtained.

**Sampling and sample size determination**: All neonates who were admitted to neonatology ward of JMC during the study period were included consecutively (using consecutive sampling technique) after obtaining written informed consent from their mothers/care takers. Sample size was calculated by using a single population proportion formula with the assumption of 95% level of confidence, 5% marginal error, taking the proportion of congenital anomalies at rate of 50%, as there is no similar study done in our setup; we added 10% non-response rate to arrive at a sample size of 422.

**Data collection method and procedures**: Structured questionnaire was developed in English after reviewing relevant literatures, translated in to two local languages (Amharic and Afan Oromo), and then back translated in to English by a third person to check for consistency. The information collected included maternal socio-demographic characteristics (age, residence, educational status, socioeconomic status, etc.), obstetric characteristics (antenatal care follow up, gravidity/parity, previous bad obstetric history, folate supplementation during pregnancy, etc.), medical history (history of drug intake during pregnancy, consanguinity, maternal acute or chronic illness, previous history of a child with congenital anomaly), maternal nutritional status(using mid upper arm circumference) and neonatal characteristics (sex, birth weight, gestational age). The data were collected by interviewing the mother/care taker and by reviewing the maternal chart (whenever available) and patient's medical records. Additionally, we demonstrated to the mothers the commonly used formulations of folic acid/iron-folate and asked them if they have received such tablets during the current pregnancy in order to minimize recall bias. Two General practitioners trained on the objectives of the study and data collection procedure collected the data. Data were collected mainly at the time of admission. Additional information was collected subsequently after admission by the time investigations like abdominal ultrasound was done. Neonates having one or more congenital anomalies detected either by clinical examination or through investigations were considered as those with congenital anomalies. The investigations were done as part of the clinical care whenever the treating physicians thought important (suspecting congenital anomalies); otherwise, the research team didn't do any of the investigations for the research purpose.

**Data processing and analysis**: Data were cleaned and coded before entering into EpiData version 4.0 and exported to the Statistical Package for Social Sciences (SPSS) version 20.0 for analysis. Descriptive statistics was carried out to see the magnitude and type of congenital anomaly. Additionally, univariate and multivariate logistic regression analysis were performed to determine factors associated with congenital anomaly. Variables with p-value of less than 0.25 at a CI of 95% on univariate logistic regression analysis were considered candidate variables for multivariate logistic regression analyses. A pvalue of less than 0.05 at a CI of 95% was taken as statistically significant.

**Ethics Approval:** Ethical clearance was obtained from the Institutional Review Board (IRB) of Jimma University Institute of Health **(Ref. No. IRB 000217/20**). Additionally, permission letter was obtained from JMC clinical director office and the department of pediatrics and child health before the commencement of the study. Written informed consent was obtained from the mother/care taker of each neonate before enrollment into the study.

## Results

A total of 422 mother-neonate pairs were approached and included into the study. Majority of the neonates were male (264, 62.6%) and born after 37 completed weeks of gestation (280, 66.4%). The mean age (and standard deviation) at presentation was 48.41 (75.80) hours with the minimum and maximum age of 5 minutes and 14 days respectively (Table 1).

**Table 1:** Characteristics of neonates admitted to neonatology ward, Jimma Medical Center, 2020 (N=422)

Variables	Category	Frequency	Percent
Gender	Male	264	62.6
	Female	158	37.4
Age at presentation	<24hr	238	56.4
	24–72hr	98	23.2
	≥72hr	86	20.4
Gestational age at birth	<37wk	142	33.6
	≥37wk	280	66.4
Birth weight	<2500gm	150	35.6
	≥2500gm	233	55.2
	Unknown	39	9.2
Type of gestation	Singleton	356	84.4
	Multiple gestation^a^	66	15.6
Place of birth	Home	22	5.2
	Institutional delivery	396	93.8
	In ambulance (on the way to health facility)	4	1.0
Mode of delivery	Spontaneous vaginal delivery	293	69.4
	Assisted delivery	13	3.1
	Cesarean section	116	27.5

a Twins (62), triplets (4)

Most of the mothers were in the age range of 19–35 years (377, 89.3%) and from rural area (257, 60.9%)(Table 2).

**Table 2:** Socio-demographic characteristics of mothers of neonates admitted to neonatology ward, Jimma Medical Center, 2020 (N=422)

Variables	Category	Frequency	Percent
Age	<19yr	19	4.5
	19–35	377	89.3
	>35	26	6.2
Ethnicity	Oromo	321	76.1
	Amhara	40	9.5
	Kaffa	29	6.9
	Yem	8	1.9
	Gurage	7	1.7
	Dawro	7	1.7
	Other^a^	10	2.4
Religion	Muslim	277	65.6
	Orthodox	94	22.3
	Protestant	50	11.8
	Catholic	1	0.2
Educational status	Can't read & write	132	31.3
	1–8	153	36.3
	9–10	73	17.3
	>10	64	15.2
Occupation	Housewife	274	64.9
	Merchant	27	6.4
	Daily laborer	6	1.4
	Farmer	67	15.9
	Employee	48	11.4
Residence	Rural	257	60.9
	Urban	165	39.1

a Siltie, Bench, Agnuak and Nuer

Only less than a quarter of the mothers (100, 23.7%) received folic acid during the current pregnancy and a third of them (147, 34.8%) were malnourished with mid upper arm circumference (MUAC) less than 23cm. Few of the mothers had previous history of a child with congenital anomalies (7, 1.7%) and first degree relative with congenital anomaly (1, 0.2%), which was cleft lip (Table 3).

**Table 3:** Obstetric and medical characteristics of mothers of neonates admitted to neonatology ward, Jimma Medical Center, 2020 (N=422)

Variables	Category	Frequency	Percent
Gravidity	1	176	41.7
	2–4	177	41.9
	≥5	69	16.4
Parity	1	193	45.7
	2–4	169	40.0
	≥5	60	14.2
Previous bad obstetric history^a^	Yes	68	16.1
	No	354	83.9
Frequency of antenatal care visit	0	17	4.0
	1–3	194	46.0
	≥4	211	50.0
Folate supplementation	Yes	100	23.7
	No	322	76.3
Maternal MUAC	<23cm	106	25.1
	≥23cm	316	74.9
Maternal chronic illness^b^	Yes	32	7.6
	No	390	92.4
Maternal substance use^c^	Yes	56	13.3
	No	366	86.7

a A mother could have more than one; Abortion (44), Early neonatal death (22), Still birth (14)

b Hypertension (20), HIV/AIDS (5), Diabetes mellitus (2), epilepsy (1), Others (bronchial asthma, schizophrenia, acute kidney injury and chronic liver disease, accounted for one each)

c Khat (47), Alcohol (9), Household exposure to tobacco smoke (4)

**Figure 1:**
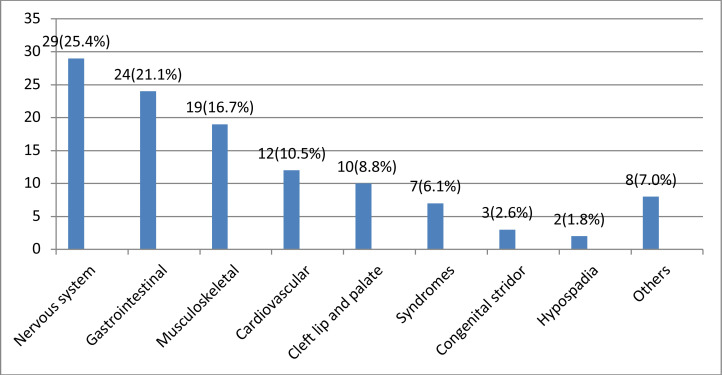
Type of congenital anomalies by system affected, neonatology ward, Jimma Medical Center, 2020 **Nervous system:** Myelomeningocele (19), Hydrocephalus (7), Microcephaly (2), Neuro-ectodermal anomally (1) **Gastrointestinal:** Anorectal anomalies (8), Tracheoesophageal fistula (4), Hirschsprung disease (3), Duodenal atresia (3), Ileal atresia (3), Omphalocele (3) **Musculoskeletal:** Congenital knee hyperextension (1), sublaxation (1), syndactyly (1), Locur button foot (1), single palmar crease (1) and Sandal toe (2) **Cardiovascular system):** Ventricular septal defect (6), Patent ductus arteriosus (2), Vascular anomally (2), Atrial septal defect (1), Transposition of great arteries (1) **Syndromes:** Down (3), Edward (1), CHARGE (1), Pierre Robin (1), VACTREL (1) **Others:** Microphthalmia (1), Atretic ear (2), ear tag (2), tongue tie (1), small mandible (1) and high arched palate (1)

Closer to one in five neonates (78, 18.5%, 95%CI 14.7–22.3) had congenital anomalies; majority (59, 13.98%) had only one type of anomaly while the rest had two (8, 1.89%) and more than two (11, 2.6%) types of anomalies. Anomalies of the nervous (29, 6.87%) and gastrointestinal (24, 5.68%) systems accounted for majority of the cases. (Figure 1) On multivariate logistic regression analysis, inadequate ANC (less than four visits) follow up (p=0.018, AOR=1.9, 95%CI1.115, 3.257) and lack of folate supplementation during pregnancy (p=0.027, AOR=2.35, 95%CI 1.101, 5.015, AOR=7.78) were associated with congenital anomalies in the neonates (Table 4).

**Table 4:** Factors associated with congenital anomaly among neonates admitted to Jimma Medical Center, 2020

Variables		Presence of congenital anomaly		p-value	AOR
				
		Yes (N, %)	No (N, %)		
**Antenatal care visits**	<4	47 (11.1)	147 (34.8)	0.02	1.9
	≥4	28 (6.6)	183 (43.5)		
Maternal Residence	Rural	57 (13.5)	200 (47.4)	0.28	1.4
	Urban	21 (5.0)	144 (34.1)		
**Folate supplementation**	Yes	9 (2.1)	91 (21.6)	0.03	2.4
	No	69 (16.4)	253 (59.9)		
Substance use	Yes	15 (3.6)	41 (9.7)	0.14	1.7
	No	63 (14.9)	303 (71.8)		
Maternal chronic illness	Yes	3 (0.7)	29 (6.9)	0.63	1.4
	No	75 (17.8)	315 (74.6)		
Maternal mid upper arm circumference	<23cm	25	81	0.19	1.5
	≥23cm	53	263		

## Discussion

In this study, we have found that around one in five neonates admitted to the neonatology ward during the study period had at least one type of congenital anomalies. This figure is significantly higher than other studies done previously in Ethiopia which have demonstrated prevalence of 5.95% in Jimma Medical Cente(10), 1.99% in selected hospitals in Addis Ababa and Amhara Region(12), and 0.62% in Felege Hiwot Hospital(14). The major reason for this difference is mainly methodological (retrospective review of registers and maternal medical records in two of the studies with possible under estimation due to incomplete or poor registration and the study population (the study was done in patients 0 to 17years old visiting the hospitals in the other). Even if variations are expected in the type of congenital anomalies depending on different factors(1,2,15), similar to other studies(4,5,7,10–14), our study demonstrated anomalies of the nervous system to be the commonest types of anomalies.

Folic acid is necessary for growth and function of human cells and is crucial for normal brain and spinal cord development during the first 4 weeks of gestation(2,4,15). Several studies have shown that folic acid reduces occurrence of different forms of congenital anomalies including neural tube defects, orofacial clefts, limb reduction defects, congenital heart defects, as well as abdominal wall defects(4,15,16). In our study as well, there was a significant association between congenital anomalies and lack of folate supplementation during pregnancy, similar to findings of other studies(4,13). This is one of the signals indicating the need to improve the quality of ANC provided at health facilities in those pregnant mothers attending ANC.

Attending antenatal care is an important component of strategies targeting reduction and early detection of congenital anomalies. By itself, just attending the ANC clinic doesn't have any effect on congenital anomalies but since during ANC visits, health education is usually given on various issues including adequate and healthy nutrition, avoidance of exposure to teratogens and different preventive, screening and curative measures are carried out, the risk of having some of the congenital anomalies can be significantly reduced. Hence, mothers with no or inadequate ANC follow up might be at risk of having newborns with congenital anomalies. Different studies(4,7) including a study done in Ethiopia before(14), have also demonstrated this, mothers with no or inadequate ANC visits having increased risk of congenital anomalies in their newborns(4,7). Our study has also demonstrated same, indicating the need of improving antenatal care attendance by pregnant mothers as early in their pregnancy course as possible and improving the quality of ANC provided by healthcare workers particularly targeting the preventive measures towards congenital anomalies.

Our study has some limitations. First, we mainly used clinical evaluation and some basic investigations (ultrasound, echocardiography) done as part of the routine care for the neonates. In order to understand the true burden of the problem, routine screening of all the neonates which we didn't do due to resource constraints is needed. Hence, we might have missed some of the congenital anomalies leading to under estimation of the magnitude of congenital anomalies. Secondly, it is carried out in a tertiary hospital serving as a referral center for several health facilities in the surrounding and hence, the magnitude of the congenital anomalies we found may not be the true reflection of the problem at all levels of health facilities and the community. Third, we enrolled only neonates admitted to the neonatology ward and didn't include the ones from labor and delivery rooms, again leading to potential over estimation of the burden. Furthermore, we didn't fully assess the factors associated with congenital anomalies like perinatal infections due to resource constraints. But despite these limitations, we feel our data contributes a lot to the scarcely available data on the problem in the low- and middle-income countries.

Congenital anomalies account for significant proportion of neonates admitted to JMC neonatology ward. There is significant association between neonatal congenital anomaly and poor antenatal attendance and periconceptional folic acid supplementation. As the burden of congenital anomaly among neonates admitted at JMC is found to be high, and since JMC is the only tertiary hospital in the area, additional attention should be given to this problem so that the facility and health workers working in the facility are well prepared to appropriately manage these neonates with congenital anomalies. Routine provision of folic acid to those attending ANC follow up needs particular attention since majority of them didn't receive it while having ANC follow up. Additionally, large, multicenter, facility as well as community-based researches should be done by involving all the stakeholders in order to understand the real burden of the problem and possible associated risk factors so that context specific preventive strategies can be put in place to reduce the burden of the problem and associated morbidities.
